# Myofibre Density Reveals a Critical Threshold Around Age 6 in Steroid‐Naïve Duchenne Muscular Dystrophy: A Retrospective Observational Study

**DOI:** 10.1111/nan.70068

**Published:** 2026-03-18

**Authors:** Tetsuhiro Yamakado, Yuka Ishikawa, Koki Ise, Lei Wang, Yoshitaka Oda, Masumi Tsuda, Taichi Kimura, Masayoshi Nagao, Manabu Ito, Shinya Tanaka, Zen‐ichi Tanei

**Affiliations:** ^1^ Department of Cancer Pathology, Faculty of Medicine Hokkaido University Sapporo Hokkaido Japan; ^2^ Department of Pathology National Hospital Organization Hokkaido Medical Center Sapporo Hokkaido Japan; ^3^ Center for Neuromuscular Disease Child Health and Development, National Hospital Organization Hokkaido Medical Center Sapporo Hokkaido Japan; ^4^ Institute for Chemical Reaction Design and Discovery (WPI‐ICReDD), Hokkaido University Sapporo Hokkaido Japan; ^5^ Department of Surgical Pathology Hokkaido University Hospital Sapporo Hokkaido Japan; ^6^ Department of Pediatrics National Hospital Organization Hokkaido Medical Center Sapporo Hokkaido Japan; ^7^ Department of Orthopedics National Hospital Organization Hokkaido Medical Center Sapporo Hokkaido Japan

**Keywords:** Duchenne muscular dystrophy, histology, histopathology, muscle biopsy, myofibre density, neuromuscular disorders, pathology

## Abstract

**Aims:**

In Duchenne muscular dystrophy (DMD), robust histological markers for assessing early disease progression remain elusive. We defined myofibre density (*MFD*) as the count of myofibres per square millimetre, which, in our preliminary survey of DMD muscle biopsies, steeply declined with age (Spearman's *ρ*: −0.85). We aimed to characterise age‐dependent *MFD* dynamics in early‐stage DMD.

**Methods:**

We retrospectively assessed 46 archival muscle‐biopsy slides (steroid‐naïve; collected > 40 years ago) using semi‐quantitative image analysis with digital restoration. *MFD* and classical histological variables were quantified. Age–*MFD* dynamics were modelled with segmented regression and validated using weakly informative Bayesian modelling. The primary measure was the *MFD* age‐breakpoint. Secondary measures included breakpoint‐detection power, age‐predictive *MFD* cut‐offs and behaviour of conventional variables across breakpoint‐defined age bands.

**Results:**

After quality and age‐distribution screening, 35 slides (age 1–11 years) were analysed. Segmented regression identified a breakpoint at 6.25 years (95% confidence interval [CI]: 5.08–7.42); after which *MFD* plateaued at lower levels. Bayesian posterior estimate was 6.37 years (95% credible interval: 5.24–7.66). A 10,000‐run Monte Carlo simulation (*n* = 35) showed approximately 80% power to recapture the breakpoint within ±1.25 years. *MFD* cut‐offs > 596 and < 426 fibres/mm^2^ corresponded to 80% and 20% probabilities of age < 6.25 years. Between the early (< 5 years) and late (≥ 7.5 years) age bands defined by the breakpoint–CI limits, myofibre‐size parameters, myofibre area and fat replacement shifted significantly.

**Conclusions:**

*MFD*, a simple metric, reveals a previously unrecognised phase of rapid myofibre loss lasting up to around age 6 in early‐stage DMD.

## Summary

- Myofibre density (*MFD*) reveals a previously unrecognised phase of rapid myofibre loss lasting up to approximately 6 years of age in patients with Duchenne muscular dystrophy (DMD) that could serve as a candidate surrogate biomarker for early disease progression.
- The identified threshold around age 6 and the observed *MFD* dynamics delineate a critical histopathological transition in disease progression, thereby providing deeper insights into the natural history of DMD.
- The original semi‐quantitative image analysis approach, incorporating novel digital restoration techniques (such as look‐up table adjustment and Focus Stacking), effectively captures early subtle tissue alterations, providing a potential framework to inform and enhance clinical decision‐making.

## Graphical abstract

We analysed archival muscle biopsies of steroid‐naïve patients with Duchenne muscular dystrophy (DMD), proposing a novel, simple metric: myofibre density (*MFD*). *MFD* sharply declines until age 6, identifying a critical threshold around this age. *MFD* provides a sensitive, reproducible biomarker for early disease progression, highlighting a potential therapeutic window and informing histopathological assessment in DMD.

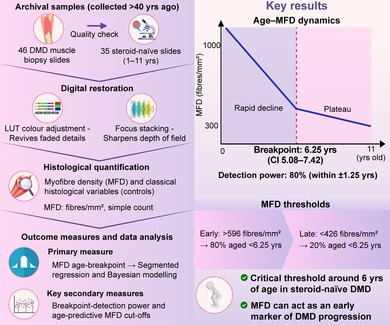

**Abbreviations**
*ABx* (parameter): age at biopsy
ANOVA: analysis of variance
ATPase: actomyosin adenosine triphosphatase
*CFA* (parameter): connective/fibrotic tissue area
CI: confidence interval
*Cov* (parameter): coefficient of variation of myofibre size
CrI: credible interval
CT: computed tomography
DMD: Duchenne muscular dystrophy
*Fat* (parameter): fatty degeneration area
FDA: Food and Drug Administration
FFPE: formalin‐fixed paraffin‐embedded
FOIs: fields of interest
G‐T: Gömöri trichrome
H&E: haematoxylin–eosin
*IntN* (parameter): internally nucleated fibre percentage per myofibre density
LOESS: Locally Estimated Scatterplot Smoothing
LOOCV: leave‐one‐out cross validation
LOOIC: leave‐one‐out information criterion
LUTs: Look‐Up Tables
*Mean* (parameter): mean myofibre size
*MFA* (parameter): myofibre area
*MFD* (parameter): myofibre density
MFS: myofibre size
MRI: magnetic resonance imaging
M‐T: Masson trichrome
*NFA* (parameter): necrotic fibre area
*Opaque* (parameter): opaque fibre percentage per myofibre density
*RFA* (parameter): regenerative fibre area
RMSE: root mean squared error
ROPE: Region of Practical Equivalence
*Sd* (parameter): standard deviation of myofibre size

## 1 Introduction

Duchenne muscular dystrophy (DMD) is a severe X‐linked neuromuscular disorder characterised by progressive skeletal muscle degeneration, ultimately leading to profound muscle weakness and cardiopulmonary complications [1, 2, 3, 4, 5]. The histopathological diagnosis of DMD relies on morphological assessment and immunohistochemical detection of dystrophin expression [6, 7, 8, 9, 10]. Despite significant advances in therapeutic interventions and disease management [1, 2, 3, 5, 11, 12, 13, 14, 15, 16, 17, 18, 19, 20, 21, 22, 23], notable gaps remain in our understanding of early‐stage DMD pathophysiology, particularly in establishing reliable histopathological markers for disease progression. Systematic investigations into histopathological correlations in DMD remain limited [6, 7, 8]. A previous report [6] suggested that histological muscle architecture remains largely unchanged until approximately 6 years of age. However, this assertion appears inconsistent with early clinical manifestations commonly observed in patients with DMD. Clinical symptoms—including stair‐climbing difficulties, waddling gait and frequent falls—typically emerge between 3 and 5 years of age [1, 4, 21], with contractures of the iliotibial bands, hip flexors and heel cords becoming evident before the age of 6 [24]. These observations suggest that histological deterioration may accelerate in a non‐linear manner—rather than remain static until a late surge—through an inflexion that conventional area‐ and fibrosis‐based metrics fail to capture. Indeed, findings on age‐related changes in myofibre and fibrotic cross‐sectional‐area metrics are scattered, leaving the age trajectory of these measures inconsistent across histopathological studies of DMD [6, 7, 8]. Here, we introduce a new metric, myofibre density (*MFD*), defined as the simple count of myofibres per square millimetre, which, in a preliminary analysis of our archival DMD muscle biopsies, showed a strong inverse correlation with patient age (Spearman's *ρ* = −0.85). These pilot data nominate *MFD* as an objective, reproducible histological marker of a previously unrecognised phase of structural loss. We, therefore, aimed to characterise the age‐dependent dynamics of *MFD*, including the possibility of a discrete breakpoint that marks the onset of tissue deterioration. To this end, we retrospectively analysed archival muscle biopsy specimens collected more than 40 years ago from steroid‐naïve patients with DMD, applying digital restoration techniques where necessary. The primary measure was to delineate the age trajectory of *MFD* and estimate whether a discrete breakpoint exists, thereby providing a histopathological benchmark for early‐stage DMD.

## 2 Materials and Methods

### 2.1 Standard Protocol Approvals, Registrations and Patient Consents

This retrospective observational study was conducted in accordance with the ethical principles outlined in the Declaration of Helsinki and approved by the central institutional review board of Hokkaido University Hospital (Approval No. 024–0310, Sapporo City, Japan), which also oversees the National Hospital Organization Hokkaido Medical Center as part of its centralised review process. Given the retrospective nature of the study, the requirement for informed consent was waived by the ethics committee.

### 2.2 Muscle Biopsy Procedures and Staining Protocols

All muscle biopsies used were performed uniformly according to guidelines described by Dubowitz and Brooke [25]. Staining protocols were standardised and performed by a single experienced laboratory technician following established muscle biopsy staining protocols developed at the National Center of Neurology and Psychiatry (Japan) during that period. At study initiation, all slides were stored together in wooden boxes in a hospital storage facility, ensuring protection from light exposure.

### 2.3 Samples

A total of 46 muscle biopsy slides obtained from the quadriceps femoris muscles of patients diagnosed with DMD between 1973 and 1984 were screened. The inclusion criteria were as follows: (1) both histopathological and clinical confirmation of DMD, with genetic verification when feasible after the introduction of genetic testing around the year 2000. (*Note*: Genetic analysis was conducted in 28 patients, with specific genotypes identified in 19. No discrepancies between initial histopathological/clinical diagnoses and subsequent genetic results were observed; the presence or absence of genetic confirmation did not affect patient inclusion or subsequent analyses. Dystrophin immunostaining, introduced at our hospital around 1990, was not performed for the cohort in this study and was not required for inclusion.); (2) patient treatment history at the former Yakumo National Hospital (subsequently integrated into Hokkaido Medical Center in 2020) from 1968 to the present; (3) absence of prior corticosteroid treatment at the time of biopsy; and (4) sufficient tissue quantity and acceptable slide quality, as assessed by three pathologists (T.Y., S.T. and Z.T.). Slides failing to meet these criteria were excluded from the analysis. Additionally, patients with Becker muscular dystrophy were excluded from the study. Archival specimens consisted of frozen‐section slides, formalin‐fixed paraffin‐embedded (FFPE) blocks, or both, depending on case availability. Histological staining techniques applied to the specimens included haematoxylin–eosin (H&E), Gömöri trichrome (G‐T) and Masson trichrome (M‐T); however, not all slides were available with each staining method (Table S1). Of the H&E, G‐T and M‐T sections, the one deemed best‐preserved by the above three pathologists was selected for image analysis. Actomyosin adenosine triphosphatase (ATPase)‐stained slides were employed not for the primary analysis but to support the statistical validation and sensitivity analyses.

### 2.4 Study Devices and Settings

Microphotographic imaging was performed using a Nikon ECLIPSE Ci microscope, equipped with a Nikon PLAN APO *λ*D 40× objective lens and a Nikon DS‐Fi3 camera, in conjunction with Nikon NIS‐D software (Nikon Corporation, Tokyo, Japan). Fields of interest (FOIs) for image analysis were jointly selected by the three pathologists. High‐resolution images were acquired at 400× magnification (2880 × 2048 pixels; resolution: 0.11 μm/pixel) and subsequently compiled into large composite images consisting of 16 tiled frames (final image resolution: 11,520 × 8192 pixels), corresponding to a 1.14‐mm^2^ field of view under a 10× objective lens. Semi‐quantitative digital image analysis was performed using Adobe Photoshop 2024 (Adobe Systems, San Jose, CA, USA), a software widely used in histopathological research [26, 27, 28, 29].

### 2.5 Morphometric Analyses and Quantifications

In this report, each parameter used in the analyses is distinctly highlighted by capitalising the initial letter and italicising it for clarity (e.g., *Parameter*).

Initially, four indices primarily assessing myofibre size (MFS) were introduced:

1. Mean MFS (*Mean*, μm^2^);
2. Standard deviation of MFS (*Sd*, μm^2^);
3. Coefficient of variation of MFS (*Cov*); and
4. Myofibre density (*MFD*, fibres/mm^2^), defined as the number of myofibres with identifiable contours per unit area, including myofibres with central nucleation or hypercontraction, but excluding necrotic and regenerating myofibres. Fibres intersecting the FOI border (i.e., with incomplete contours at the image edge) were excluded from the *MFD* tally to address potential edge effects.

Additionally, seven conventional morphometric parameters, established in previous studies [6, 7, 30], were analysed, each expressed as a percentage per FOI unless otherwise specified:

1. Myofibre area (*MFA*), measured irrespective of pathological features;
2. Connective/fibrotic tissue area (*CFA*);
3. Necrotic fibre area (*NFA*);
4. Regenerative fibre area (*RFA*);
5. Fatty degeneration area (*Fat*);
6. Opaque (hypercontracted) fibre percentage per *MFD* (*Opaque*, count/*MFD*, %); and
7. Internally nucleated fibre percentage per *MFD* (*IntN*, count/*MFD*, %).

**FIGURE 1:**
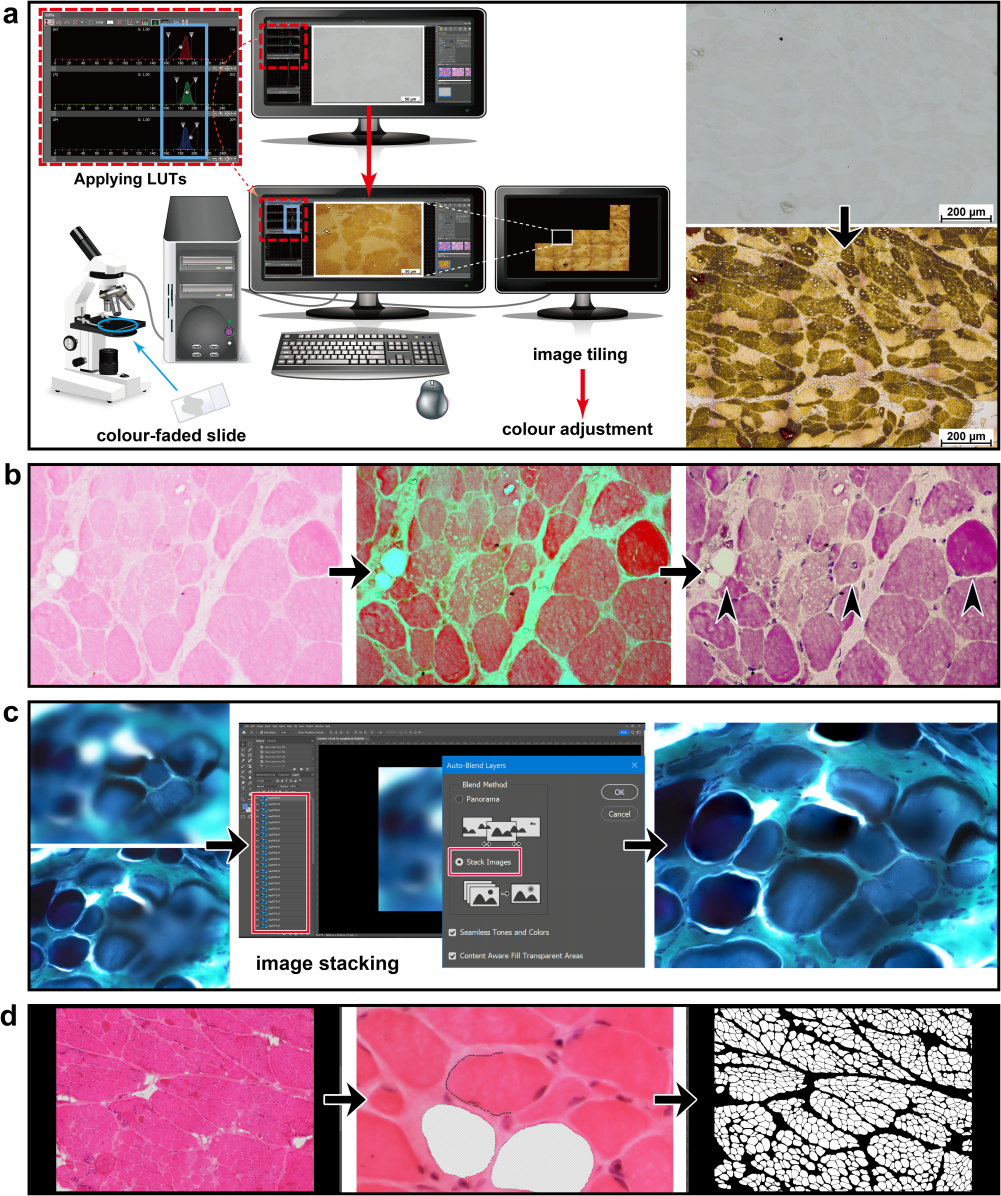
Sequential workflow for progressive restoration and semi‐quantitative image analysis. (a) This panel illustrates the application of LUTs to digitally restore a severely faded ATPase‐stained specimen (pH 9.4), representing the most extensively degraded example in our series. Adjusting LUTs in the live camera preview mode enhances the visibility of tissue structures that have faded over time. The processed images are subsequently tiled to generate a high‐resolution, composite image encompassing the entire FOI. Next, commonly used colour adjustment functions in Photoshop (e.g., colour balance and hue/saturation) effectively restore an ATPase‐like appearance. (b) The restoration approach described in Panel (a) is adapted for H&E staining, in which nuclear structures are difficult to discern as a result of fading. Initially, LUTs are applied, rendering otherwise undetectable nuclei in green, thereby improving the visualisation of nuclear morphology and myofibre boundaries. Subsequently, colour adjustment functions in Photoshop are employed to transform the green‐highlighted nuclei into a purple hue, thereby restoring an appearance characteristic of H&E staining. This approach facilitates the clear identification of features such as centrally nucleated and hypercontracted myofibres, as well as areas of fatty infiltration, which become clearly discernible (arrowheads). (c) Focus Stacking is employed to enhance the visualisation of a G‐T‐stained specimen. Multiple images are captured at varying focal depths and subsequently combined into a single, sharply focused image using the Auto‐Blend Layers function in Adobe Photoshop. (d) This panel demonstrates the use of the Magnetic Lasso Tool in Adobe Photoshop to delineate individual myofibres in an H&E‐stained specimen. The segmented image is subsequently converted into a monochrome format to simplify visualisation and facilitate quantitative analysis. *Note: The illustration in panel a was generated using Adobe Illustrator software (Adobe Systems, San Jose, CA, USA) and incorporates freely available resources from Pixabay* (https://pixabay.com) *alongside screenshots of the Nikon NIS‐D software interface (Nikon Corporation, Tokyo, Japan)*. *This figure also includes screenshots of Adobe Photoshop (Adobe Systems, San Jose, CA, USA), which was used in the image analysis workflow (panels c and d).*Abbreviations: ATPase, actomyosin adenosine triphosphatase; FOI, field of interest; G‐T, Gömöri trichrome; H&E, haematoxylin–eosin; LUTs, look‐up table; *MFA*, myofibre area; MFS, myofibre size.

To enhance image quality and facilitate accurate morphometric analysis, colour restoration was performed using Look‐Up Tables (LUTs) in Nikon NIS‐D software, a tool commonly employed in fluorescence and confocal microscopy [31, 32]. High‐resolution images at 400× magnification were acquired in real‐time using LUTs and subsequently tiled to generate a large composite image (Figure 1a). LUTs not only facilitated the identification of internal nuclei and opaque fibres but also clearly delineated myofibre boundaries, fatty infiltration and other histopathological alterations (Figure 1b). To mitigate artefacts arising from focal plane variations, a known limitation in high‐magnification imaging, a Focus Stacking approach [33] was implemented using an automated script in Adobe Photoshop. This technique combines multiple images captured at varying focal depths (Figure 1c), yielding an extended depth‐of‐field image that is then integrated into the composite large image. Artefactual regions that could not be rectified using these methods were excluded from the FOI. Myofibre tracing was performed using the Magnetic Lasso Tool in Adobe Photoshop (contrast setting: 40, point frequency: 100) (Figure 1d). To ensure precise quantification, MFS‐related parameters were analysed using a monochrome‐converted version of the large image, enhancing contrast and facilitating accurate measurement (Figure 1d). Individual myofibre areas were quantified using the Analysis function in Adobe Photoshop, in which each myofibre was independently delineated to prevent measurement errors arising from overlapping or contiguous fibres. The function assigned unique labels to each selected region of interest, which were subsequently used to compute all parameters following the methodology previously described. Morphometric data extracted from Adobe Photoshop were exported as text files, further processed in Microsoft Excel, and saved in CSV format for statistical analysis described in the following section.

### 2.6 Statistical Analyses

All computations were performed in R 4.3.2; the complete datasets and scripts are provided as Data S1 and S2. A significance threshold of *p* < 0.05 was applied to all statistical tests.

#### 2.6.1 Preliminary Correlation Screening

Spearman's rank correlation coefficient (*ρ*) was used to explore the monotonic association of each histomorphometric variable with age at biopsy (*ABx*).

#### 2.6.2 Primary Measure: Segmented Regression and Bayesian Modelling

Based on this screening, the statistical analysis plan was finalised. The primary measure was the age‐dependent breakpoint (*ψ*) in *MFD* and its associated slopes (*β*_1_, *β*_2_). *MFD* was modelled with a segmented regression (Muggeo 2003 [34]) as follows:

$$y_{i}=\alpha +\beta_{1}{\left(x_{i}-\psi \right)}_{-}+\beta_{2}{\left(x_{i}-\psi \right)}_{+}+\varepsilon_{i},\;\varepsilon_{i}\sim N\left(0,\sigma^{2}\right)$$

Segmented regression was applied to patients < 11 years (see Section 3). *ψ* was visually initialised [34] using a moving‐average plot of *MFD* and compared with a single‐slope model by an *F*‐test within an analysis of variance (ANOVA) framework. Model fit was quantified by the leave‐one‐out cross‐validated root‐mean‐squared error (LOOCV‐RMSE). Robustness was assessed with a weakly informative Bayesian model, whose priors were centred on the segmented regression estimates. In this model, *x*_i_* served as the predictor and the same variable (*y*_*i*_) as the response:

$$y_{i}=\alpha +\beta_{1}{x_{i}}^{*}+\beta_{∆}\left({x_{i}}^{*}-\psi^{*}\right)\cdot \text{step}\left({x_{i}}^{*}-\psi^{*}\right)+\varepsilon_{i},\;\varepsilon_{i}\sim N\left(0,\sigma^{2}\right)$$

$$\psi^{*}:\text{posterior breakpoint mode}$$

$\mathrm{\mu x}=\text{mean}\;\mathrm{ABx}\;\left(<11\;\text{years}\right),\;{x_{i}}^{*}=x_{i}-\mathrm{\mu x},\;\psi^{*}=\psi -\mathrm{\mu x}$

*β*_1_ and *β*_Δ_ were tested against a region of practical equivalence (ROPE) derived from the paired measurement error between the section used for *MFD* quantification (H&E, G‐T or M‐T, as applicable) and its ATPase‐stained counterpart. Predictive adequacy was compared using the leave‐one‐out information criterion (LOOIC).

#### 2.6.3 Secondary Measures: Power Estimation, Age Cut‐Offs and Variance Analysis

(1) A 10,000‐run Monte Carlo resampling, parameterised by the LOOCV‐RMSE, estimated the sample size required to recapture *ψ* within ±1.0–1.5 years. (2) A single‐predictor logistic model translated *MFD* into age‐probability cut‐offs (80%, 50% and 20%). (3) To test cross‐parameter validity, a group‐wise variance analysis was performed. Age strata were defined by the 95% confidence interval (CI) of the *MFD* breakpoint: Early (< lower CI limit), Transitional (CI span) and Late (≥ upper CI limit). Normality (Shapiro–Wilk) and homogeneity (Levene) were assessed using one‐way ANOVA with Tukey's post hoc test, or Kruskal–Wallis with Conover's post hoc test. In line with the recent guidance [35], we calculated effect sizes—Hedges' *d* or Cliff's *δ*—with their 5000‐bootstrap 95% CIs.

## 3 Results

### 3.1 Patient Selection

From an initial cohort of 46 muscle biopsy slides, 38 samples (aged 1–16 years) were selected after quality screening (Table 1). A preliminary correlation analysis was performed on these 38 slides. Because there were only 3 samples in 11–16 years, an age‐distribution screening was applied for the main segmented regression analysis, yielding 35 samples aged 1–11 years.

**TABLE 1:** Summary of parameters, biopsy year and genotype information.

Participant	*ABx* ^a^	Bx. year	*Mean*	*Cov*	*Sd*	*MFD*	*MFA*	*Fat*	*CFA*	Genotype^b^
1	1.0	1983	457.9	0.58	265.7	1471.9	68.9%	0.2%	29.6%	NA
2	1.9	1977	565.3	0.48	271.9	1227.0	72.3%	0.1%	26.7%	NA
3	2.0	1975	622.1	0.53	328.7	1069.5	68.7%	0.2%	30.7%	exon44–47 del
4	2.4	1979	598.1	0.56	336.2	1149.8	71.6%	1.7%	25.8%	exon56 del
5	3.3	1977	851.2	0.54	455.7	656.8	57.3%	0.7%	39.2%	—
6	3.7	1975	531.3	0.44	230.9	953.5	53.7%	0.2%	45.7%	exon44–47 del
7	4.0	1984	1037.9	0.53	551.2	563.2	53.5%	1.3%	41.6%	—
8	4.1	1979	577.5	1.20	693.4	867.2	53.4%	5.3%	40.3%	exon 44–51 del
9	4.3	1973	828.5	0.50	417.0	766.0	65.7%	0.6%	32.1%	exon56 del
10	4.6	1984	870.4	0.65	568.3	545.5	45.9%	1.7%	48.9%	exon44 del
11	4.7	1978	721.1	0.51	365.7	815.2	62.1%	0.8%	36.7%	exon36 del
12	5.0	1980	1007.4	0.50	503.6	571.1	59.5%	1.7%	37.3%	exon43–50 del
13	5.2	1980	624.3	0.53	330.3	976.4	64.9%	0.1%	34.0%	—
14	6.0	1975	725.1	0.82	591.6	552.0	38.5%	1.4%	58.7%	—
15	6.2	1977	1352.0	0.43	585.0	454.7	65.8%	0.1%	31.6%	—
16	6.2	1977	1176.2	1.11	1309.4	365.8	42.2%	1.4%	54.6%	NA
17	6.2	1978	556.3	0.88	489.5	559.9	29.2%	0.7%	67.1%	exon20, exon59 Mut
18	6.3	1979	1602.3	0.78	1244.7	300.7	51.7%	3.5%	44.4%	NA
19	6.4	1979	1520.6	0.95	1442.2	343.8	54.7%	3.1%	40.7%	NA
20	6.5	1979	1554.0	0.61	954.3	400.7	66.8%	0.5%	31.7%	exon6–8 del
21	6.6	1983	1683.7	0.70	1176.0	216.6	36.3%	1.5%	59.9%	—
22	7.0	1978	850.3	0.73	623.7	553.8	47.1%	3.9%	45.3%	—
23	7.2	1979	680.0	0.71	479.3	572.7	39.8%	13.2%	46.6%	exon46–48 del
24	7.2	1976	970.2	0.98	954.0	445.2	45.8%	3.6%	50.2%	NA
25	7.8	1980	772.0	0.60	464.8	458.1	36.4%	6.1%	57.0%	NA
26	8.3	1980	1231.8	1.72	2118.5	245.4	30.4%	6.4%	62.4%	NA
27	8.7	1982	1048.4	1.26	1324.3	497.9	53.9%	0.05%	41.7%	exon50 del
28	8.8	1981	1327.3	0.82	1081.8	282.9	38.6%	3.5%	55.0%	exon3 del
29	8.9	1979	613.6	0.86	526.5	386.5	23.5%	18.6%	55.9%	—
30	9.1	1982	1285.4	0.95	1215.0	423.0	59.0%	5.0%	33.6%	exon44 del
31	9.7	1976	889.0	0.75	666.2	291.0	25.4%	3.2%	68.2%	—
32	9.8	1982	1203.7	0.78	943.5	414.1	52.8%	8.6%	37.3%	exon51–60 del
33	10.3	1980	3643.5	0.99	3603.9	143.9	58.2%	10.3%	30.8%	exon12 dupl
34	10.7	1982	909.6	0.90	821.4	240.5	20.8%	6.0%	71.6%	exon44 del
35	10.9	1977	1565.2	0.77	1199.8	393.5	67.7%	3.0%	28.7%	exon56 del
36	12.8	1983	1028.5	1.07	1096.0	219.3	20.9%	3.4%	73.7%	NA
37	15.1	1973	1953.9	0.92	1787.6	170.2	33.9%	9.4%	54.6%	NA
38	16.3	1973	1504.0	1.46	2189.0	172.1	26.5%	15.4%	55.9%	exon46–49 del

Abbreviations: *ABx*, age at biopsy; Bx, biopsy; *CFA*, connective/fibrotic tissue area; *Cov*, coefficient of variation of myofibre size; del, deletion; dupl, duplication; *Fat*, fatty degeneration area; *Mean*, mean myofibre size; *MFA*, myofibre area; *MFD*, myofibre density; MLPA, multiplex ligation‐dependent probe amplification; Mut, mutation; NA, not available; PCR, polymerase chain reaction; *Sd*, standard deviation of myofibre size.

^a^ *ABx* was treated as a continuous variable in correlation analyses and is reported to one decimal place. For analytical precision, the exact age was determined by incorporating the month of biopsy, determined by dividing a year into 12 and adding the resulting fraction to the *ABx*. For patients with identical age and month values, the ranking was further refined by accounting for the number of weeks.

^b^ Genetic mutations were identified via MLPA or PCR. ‘NA’ denotes patients who did not undergo genetic testing, whereas an em dash (—) indicates cases in which no mutation was identified despite testing. Genetic results are provided primarily to confirm diagnostic validity and for descriptive reference.

### 3.2 Histopathological Findings

**FIGURE 2:**
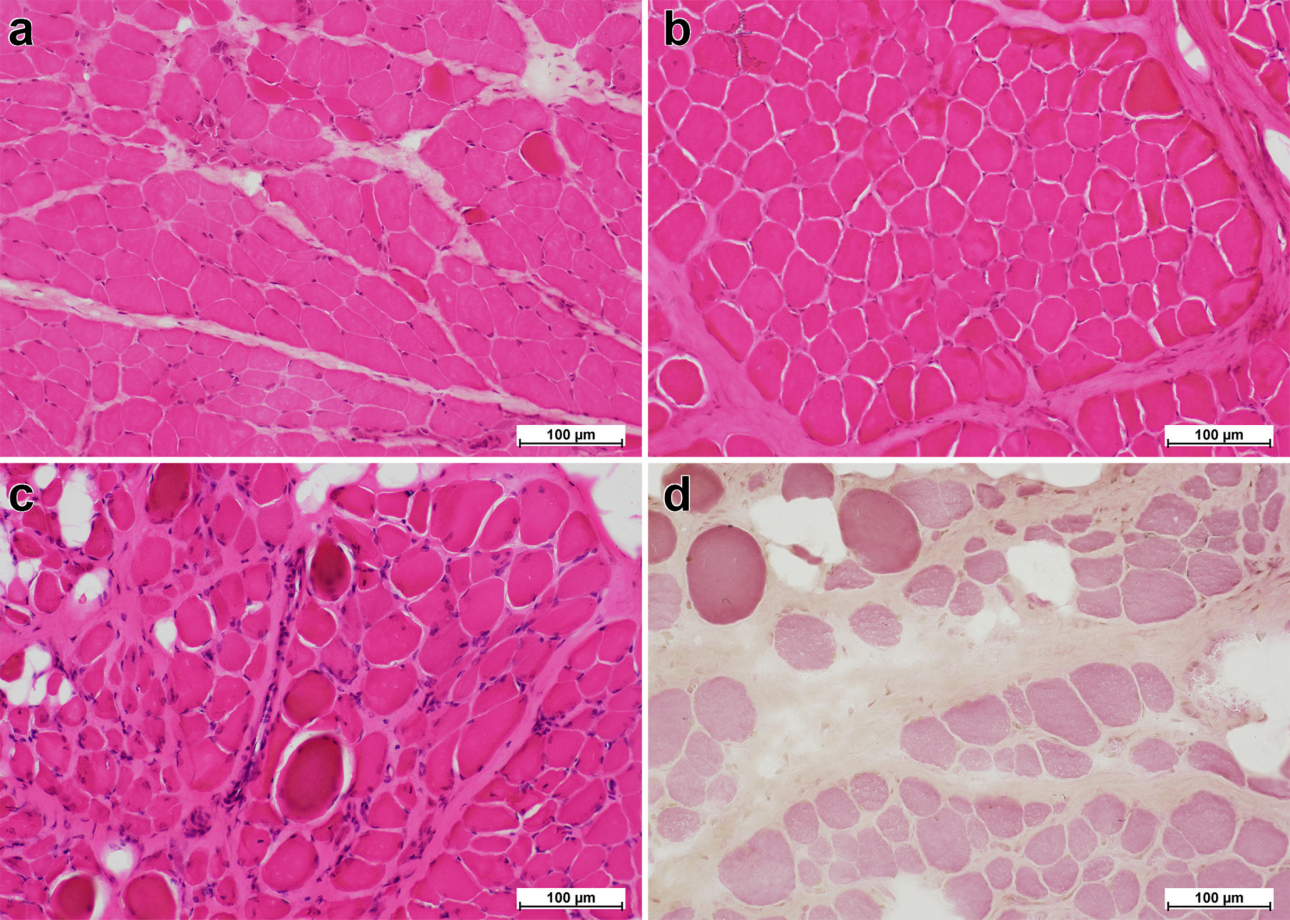
Representative H&E‐stained images of patients with DMD of different ages. Each panel presents representative H&E‐stained images obtained from patients of specific ages (200× magnification for each): 4 years and 4 months (a), 6 years and 2 months (b), 7 years and 0 months (c) and 9 years and 9 months (d). (a) In the 4‐year‐old patient, myofibres exhibit uniform morphology with well‐preserved *MFA* and an increased presence of connective tissue. (b) The 6‐year‐old patient similarly demonstrates uniform myofibres with *MFA*; however, a substantial reduction in *MFD* is observed upon quantification. Comparison of Panels (a) and (b) reveals *MFA* values of 65.69% and 65.77%, respectively, while *MFD* values decline from 765.96 to 454.67 fibres/mm^2^. Connective tissue is not increased, with *CFA* values of 32.06% and 31.55% in Panels (a) and (b), respectively. (c) In the 7‐year‐old patient, increased variability in MFS is evident, accompanied by pronounced proliferation of connective tissue and the emergence of mild fat infiltration. These morphological alterations are more pronounced compared with patients younger than 6 years. (d) The 9‐year‐old patient exhibits significant reductions in both *MFD* and *MFA*, accompanied by extensive proliferation of connective tissue and localised fat infiltration. The FOI is captured using a 10× objective, while all panels are displayed at 20× magnification. Due to haematoxylin fading, nuclei and inflammatory cells are difficult to discern, necessitating LUTs adjustment. Abbreviations: *CFA*, connective/fibrotic tissue area; DMD, Duchenne muscular dystrophy; FOI, fields of interest; H&E, haematoxylin‐eosin; LUTs, look‐up tables; *MFA*, myofibre area; *MFD*, myofibre density; MFS, myofibre size.

Histological evaluation revealed distinct differences in *MFD* patterns between specimens obtained from patients younger than approximately 6 years and those from older age groups, as described in subsequent sections. Most samples exhibited characteristic histological alterations, including MFS variation, the presence of hypercontracted myofibres and internally nucleated myofibres, an increase in connective tissue, reduced *MFA*, widened inter‐myofibre spacing and fatty degeneration (Figure 2).

### 3.3 Correlations Between *ABx* and Histological Parameters

**FIGURE 3:**
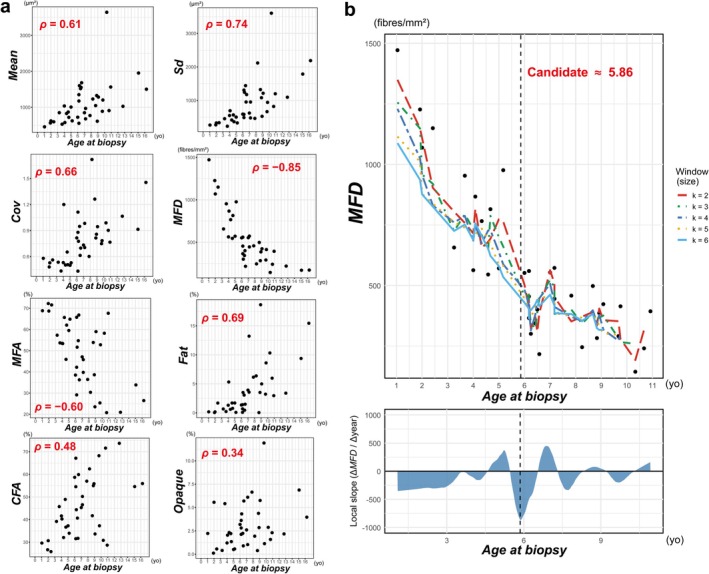
Correlation analysis and mean‐average plot. (a) Scatter plots illustrate the relationships between *ABx* and each parameter demonstrating a statistically significant correlation, with Spearman's correlation coefficients (*ρ*) indicated. Each data point represents an individual sample. (b) The upper panel displays the moving‐average curve (window size, *k* = 2–6) and the fitted LOESS regression curve for *MFD* in relation to *ABx*. The LOESS smoothing span (0.30) was objectively determined by minimising RMSE compared with the moving average (*k* = 2). The dashed vertical line indicates the steepest decline identified through LOESS regression analysis, corresponding to a candidate breakpoint at 5.86 years. Individual data points represent actual patient measurements. The lower panel illustrates the local slope derived from the LOESS curve, which has been smoothed further with half of the optimal span. Abbreviations: *ABx*, age at biopsy; *CFA*, connective/fibrotic tissue area; *Cov*, coefficient of variation of myofibre size; *Fat*, fatty degeneration area; *Mean*, mean myofibre size; LOESS, Locally Estimated Scatterplot Smoothing; *MFA*, myofibre area; *MFD*, myofibre density; *Opaque*, percentage of opaque fibres relative to *MFD*; RMSE, root mean squared error; *Sd*, standard deviation of myofibre size.

Correlations between *ABx* and histological parameters were assessed. All variables contained complete datasets. The results of image‐based quantitative analyses are presented in Table 1, alongside biopsy year and genotype data. Statistically significant Spearman's correlation coefficients for histological parameters were as follows: *Mean* (*ρ* = 0.61), *Sd* (*ρ* = 0.74), *Cov* (*ρ* = 0.66), *MFD* (*ρ* = −0.85), *MFA* (*ρ* = −0.60), *Fat* (*ρ* = 0.69), *CFA* (*ρ* = 0.48) and *Opaque* (*ρ* = 0.34) (Figure 3a). No significant correlations were observed for *IntN*, *NFA*, or *RFA* (additional data are given in Figure S1 and Data S2).

### 3.4 Segmented Regression Analysis

**FIGURE 4:**
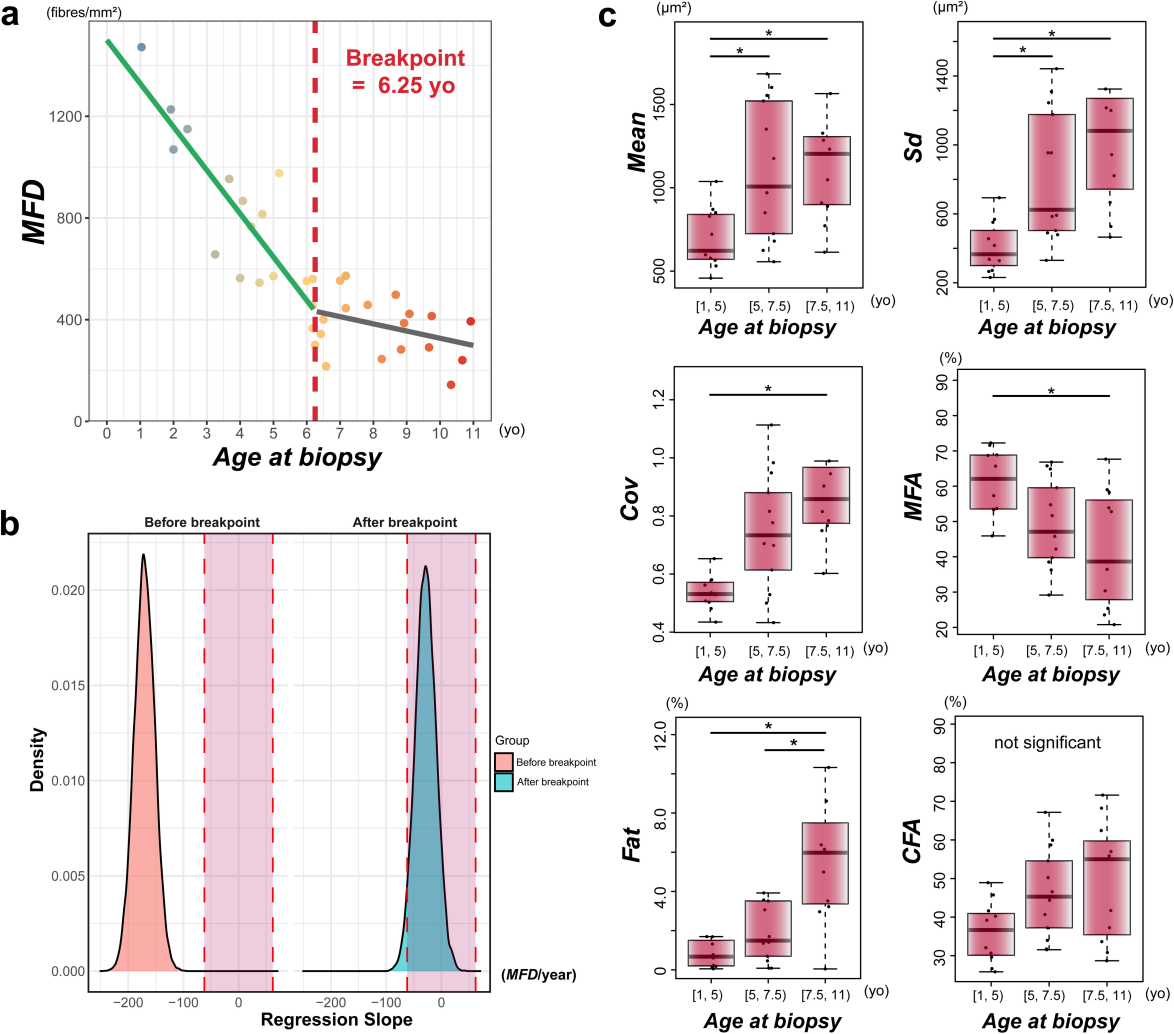
Segmented regression, Bayesian modelling and variance analysis. (a) Segmented regression analysis demonstrates a statistically significant breakpoint at age 6.25 years (vertical dashed line). A rapid decline in *MFD* is evident before this age, with a subsequent plateauing of the rate of decline afterwards. (b) This plot presents the posterior distributions of the regression slopes obtained from the Bayesian segmented regression analysis, divided by the breakpoint. The left density plot (red) represents the posterior distribution of slopes before the breakpoint, while the right plot (blue) corresponds to slopes after the breakpoint. Dashed red lines indicate the ROPE (±62.00 *MFD*/year). (c) Box plots illustrate the results of variance analyses and corresponding post hoc comparisons for multiple morphological parameters: *Mean*, *Sd*, *Cov*, *MFA*, *Fat* and *CFA*. Data are stratified by three specific age groups: 1–5, 5–7.5 and 7.5–11 years. The age groups are denoted using the interval notation [*X*, *Y*), indicating that each range includes ages greater than or equal to *X* and strictly less than *Y*. Statistical comparisons between groups were performed using ANOVA and the Kruskal–Wallis test. Statistically significant differences identified by these pairwise comparisons are indicated by asterisks (*) adjusted by the false‐discovery‐rate method. Outliers are not displayed in the plots to maintain visual clarity; however, they were included in all statistical analyses and calculations. Abbreviations: ANOVA, analysis of variance; *CFA*, connective/fibrotic tissue area; *Cov*, coefficient of variation of myofibre size; *Fat*, fatty degeneration area; *Mean*, mean myofibre size; *MFA*, myofibre area; *MFD*, myofibre density; ROPE, Region of Practical Equivalence; *Sd*, standard deviation of myofibre size.

Because only three quality‐qualified biopsies came from patients aged 11–16 years, segmented regression was limited to cases under 11 years (*n* = 35). The initial breakpoint was estimated at 6 years (Figure 3b). The segmented regression identified a breakpoint at 6.25 years [95% CI: 5.08, 7.42] for *MFD* (Figure 4a). Before this point, *MFD* declined steeply and significantly, with a slope of −170.68 fibres/mm^2^ per year [95% CI: −213.41, −127.95]. After this point, the slope was −28.31 fibres/mm^2^ per year [95% CI: −76.47, 19.85] and was not statistically significant. This analysis yielded an adjusted *R* ^2^ of 0.80. ANOVA comparing the simple linear regression with the segmented regression showed a significantly improved model fit for the segmented model (*F*[2,31] = 10.37; *p* < 0.001). Additionally, varying the initial breakpoint seed from 4 to 8 years in 0.5‐year increments consistently returned the same estimated breakpoint of 6.25 years (maximum deviation < 0.003 years). Meanwhile, segmented regression of the other parameters—*Mean*, *Sd*, *Cov*, *MFA*, *Fat*, *CFA* and *Opaque*—yielded no statistically significant improvement in model fit, regardless of the initial breakpoint setting.

### 3.5 Bayesian Modelling

The Bayesian posterior analysis of the *MFD* segmented regression model estimated a breakpoint at 6.37 years (95% credible interval [CrI]: 5.24, 7.66) (Table 2). Posterior slope estimates indicated a significant decline of −165.14 fibres/mm^2^ per year [95% CrI: −200.32, −131.15] before the breakpoint and a substantially lower decline of −28.72 fibres/mm^2^ per year [95% CrI: −67.84, 15.94] without statistical significance after the breakpoint (Table 2). Detailed Markov chain Monte Carlo convergence diagnostics and posterior summary statistics are provided in Data S2. Bayesian model comparison using LOOIC indicated that the segmented model had better predictive performance than a simpler linear model, with an expected log predictive density difference of 7.31. The mean absolute difference between paired original and ATPase‐stained *MFD* values was 62.00 fibres/mm^2^, which was used to set the ROPE. The posterior slope before the breakpoint was completely outside this ROPE interval (0% overlap; Figure 4b). In contrast, the slope after the breakpoint overlapped the ROPE interval (95.01% of posterior distribution within ROPE; Figure 4b). Sensitivity analyses showed that widening the ROPE by +1, +1.5 and +2 standard deviations of the paired‐difference distribution produced the same pattern (Data S2): the post‐breakpoint slope consistently had ≥ 95% (up to 100%) of its posterior mass inside every expanded interval, whereas the pre‐breakpoint slope remained almost entirely outside (≤ 5%).

**TABLE 2:** Bayesian segmented regression model with weakly informative priors^a^.

Parameter	Mean	SD	2.5% CrI	Median	97.5% CrI
Intercept (pre‐BP)	1459.61	81.67	1292.30	1460.28	1617.48
Slope (pre‐BP)^b^	−165.14	17.34	−200.32	−164.87	−131.15
Slope (post‐BP)^b^	−28.72	21.09	−67.84	−29.73	15.94
Breakpoint age (years)	6.37	0.58	5.24	6.37	7.66

Abbreviations: *ABx*, age at biopsy; BP, breakpoint; CrI, credible interval; *MFD*, myofibre density; ROPE, Region of Practical Equivalence; SD, standard deviation.

^a^ In this Bayesian model, *ABx* centred at the mean (*x*_i_*) was modelled as the predictor variable, and the *MFD* (*y*_*i*_) as the response variable (as shown in the main text): $y_{i}=\alpha +\beta_{1}\left({x_{i}}^{*}-\psi^{*}\right)+\beta_{∆}\left({x_{i}}^{*}-\psi^{*}\right)\cdot \text{step}\left({x_{i}}^{*}-\psi^{*}\right)+\varepsilon_{i},\;\varepsilon_{i}\sim N\left(0,\sigma^{2}\right)$ Weakly informative priors were set as follows (based on original segmented model): *α* (intercept): normal(1500, 300); *β*_1_ (slope pre‐breakpoint): normal (−171, 40); *β*_Δ_ (difference in slope): normal(142, 40); ψ* (breakpoint age; defined at zero on centred scale): normal (0, 2).

^b^ The fraction of posterior distribution within the ROPE was 0% for the pre‐breakpoint slope, indicating robust statistical support for a non‐zero slope before the breakpoint, and 95.01% for the post‐breakpoint slope, showing practical equivalence to zero.

### 3.6 Power Estimation (Monte Carlo Simulations)

The sample‐size simulations using the Monte Carlo method indicated that our current sample size (*n* = 35) yielded a 72.43% probability of detecting the breakpoint within ±1.00 years, 79.47% within ±1.25 years, and 84.54% within ±1.5 years (Table 3). Probabilities for other sample sizes are also summarised in Table 3.

**TABLE 3:** The results of sample‐size simulations by Monte Carlo method^a^.

Sample size	Precision (±1 year)^b^	Precision (±1.25 years)^b^	Precision (±1.5 years)^b^
20	58.18%	65.43%	72.66%
25	65.65%	71.01%	76.77%
30	69.80%	76.43%	82.08%
***35***	***72.43%***	***79.47%***	***84.54%***
40	75.11%	81.64%	86.44%
45	77.00%	84.08%	88.16%
50	79.71%	86.14%	89.96%
55	81.59%	87.63%	91.58%
60	83.42%	89.01%	92.40%
65	84.51%	89.81%	93.87%
70	85.61%	90.65%	94.01%

Note: The rows highlighted in bold represent the sample sizes corresponding to those in our current study.

^a^ In each replicate, age at biopsy (*ABx*) values were bootstrapped from the observed < 11 years cohort, while model parameters (*β*_1_, *β*_Δ_, *σ*, *ψ*) were resampled from the Bayesian posterior (*n* = 10,000 draws). A run was counted as successful when the segmented‐regression breakpoint lay within ±1, 1.25 and 1.5 years of the true *ψ*.

^b^ Precision columns report the Monte Carlo probability that the breakpoint estimate falls within ±1.0, 1.25 or 1.5 years of the posterior‐sampled true breakpoint (see also Data S2).

### 3.7 Age Cut‐Offs (Logistic Regression Analysis)

Logistic regression analysis using the breakpoint age of 6.25 years identified three distinct *MFD* zones associated with differing probabilities of cases being younger than the breakpoint (Table 4). Specifically, *MFD* > 595.51 fibres/mm^2^ corresponded to a high probability (> 80%, 10 cases), 426.36–595.51 fibres/mm^2^ indicated an intermediate probability (20%–80%, 11 cases), and *MFD* < 426.36 fibres/mm^2^ was associated with a low probability (< 20%, 14 cases).

**TABLE 4:** Thresholds derived from logistic regression model (breakpoint = 6.25 years old).

*MFD* zone (fibres/mm^2^)	Cases	Probability (< breakpoint)	Hypothetical therapeutic window^a^
> 595.51	10	> 80%	Large therapeutic window
426.36–595.51	11	20%–80%	Requires individual judgement
< 426.36	14	< 20%	Small therapeutic window

Abbreviation: *MFD*, myofibre density.

^a^ This therapeutic window is hypothetical (or theoretical), estimated from probabilities derived from logistic regression.

### 3.8 Group‐Wise Variance Analysis

Group‐wise variance analysis showed significant pair‐wise differences in *Mean*, *Sd* and *Fat* between the Early band and each older band, a significant difference in *MFA* and in *Cov* only between Early and Late (Figure 4c). In the Early–Late comparison, Cliff's *δ* indicated large effects for *Mean* (*δ* = 0.80 [95% CI: 0.49, 1.00]), *Sd* (*δ* = 0.88 [95% CI: 0.65, 1.00]), *Cov* (*δ* = 0.83 [95% CI: 0.49, 1.00]) and *Fat* (*δ* = 0.75 [95% CI: 0.36, 1.00]), while Hedges' *d* showed a pronounced decrease in *MFA* (*d* = −1.36 [95% CI: −2.54, −0.65]) and a large‐magnitude increase in *CFA* (*d* = 1.04 [95% CI: 0.31, 2.16]); however, *CFA* did not attain statistical significance in the variance analysis. Full statistics, with *MFD* itself included as a positive control, are provided in Table S2: Although recent guidelines caution against using *p* values as evidence of statistical significance [35], we evaluated statistical significance based on false‐discovery‐rate–adjusted *p* values because our variance analyses involved multiple comparisons.

## 4 Discussion

Our findings demonstrate a steep decline in *MFD* before the 6.25‐year breakpoint, revealing a previously unrecognised phase of rapid myofibre loss in the early stages of DMD. Two key novel insights emerged from our analysis: (1) *MFD* shows promise as a candidate surrogate biomarker for early‐stage DMD progression and (2) the identified age 6 threshold and *MFD* dynamics delineate a critical histopathological transition in disease pathology. By capturing early tissue alterations that are undetectable through conventional indices, our results offer a robust framework for histological monitoring and may provide the pathological evidence needed to refine age‐based therapeutic guidelines.

Morphologically, MFS assessment has often relied on semi‐quantitative scoring and/or limited sampling [6, 7]. In this study, because a completely objective assessment of MFS necessitates the enumeration of all individual myofibres, *MFD* values inherently emerged as part of our quantitative analytical framework. Notably, despite comparable *MFA*, a 1.5‐fold difference in *MFD* was observed between patients aged 4 and 6 years (765.96 vs. 454.67 fibres/mm^2^; Figure 2a,b), underscoring a substantial histopathological distinction. These observations reveal that area‐based quantification can mask early histopathological changes, whereas density‐based measures more sensitively capture such alterations. Microscopically, densely packed myofibres may visually convey an impression of high *MFD* value. However, our data suggest that mild hypertrophy may contribute to a uniform yet globally enlarged myofibre morphology that could obscure early‐stage myofibre depletion upon cursory examination—a change that may underlie the calf muscle hypertrophy in patients with DMD. The stability of MFS‐related parameters before age 5, particularly the narrow interquartile range in *Cov* (0.51–0.57; Table 1 and Figure 4c), further supports these interpretations. Additionally, Bell and Conen (1967) reported a sharp rise in MFS variability at 5–7 years, a pattern that agrees with the MFS trajectories we observed, and they likewise linked this morphological shift to the calf muscle hypertrophy [36]. These findings indicate that the structural transitions captured by *MFD* and those captured by MFS are complementary. The concurrence of these inverse inflexion patterns within the same 5‐ to 7‐year interval suggests that compensatory myofibre hypertrophy masks myofibre loss until approximately 6 years of age, after which the regenerative reserve may be exhausted and myofibre depletion may become morphologically overt.

In our statistical analysis, all parameters, including *ABx*, were treated as continuous variables to quantitatively assess their relationships. Spearman's correlation analysis initially revealed a strong inverse correlation between *ABx* and *MFD*. Segmented regression confirmed a distinct breakpoint at 6.25 years. This analysis revealed a steep, statistically significant decline in *MFD* before the breakpoint (< 6.25 years), whereas the post‐breakpoint slope flattened, indicating a marked deceleration of myofibre loss after this point. Bayesian re‐estimation corroborated the breakpoint, and Monte Carlo power analysis showed that the current sample size (*n* = 35) gives approximately 80% probability of recapturing this breakpoint within ±1.25 years of age, indicating the robustness of our findings. The age cut‐offs and their *MFD* thresholds identified by logistic modelling possibly represent hypothetical therapeutic windows (Table 4), and their clinical validity should be evaluated in future research. A group‐wise variance analysis demonstrated significant shifts in several classical histological variables—except for *CFA*—when stratified by the breakpoint‐derived age bands. This validates the cross‐parameter applicability of *MFD*‐based stratification and partially accords with earlier studies [6, 8, 36]. The large effect sizes observed between the Early and Late bands for MFS‐related parameters (*Mean*, *Sd* and *Cov*) are noteworthy.

We propose that around age 6 represents a histopathological critical threshold in the progression of DMD, coinciding with a peak in early muscle deterioration as indicated by a marked reduction in *MFD*. This age‐linked drop suggests that *MFD* serves as a sensitive histological marker for early disease dynamics. While it has been posited that the pathophysiology of DMD is driven by recurrent cycles of necrosis and regeneration, ultimately culminating in an incomplete compensatory process [6, 37], our findings indicate that a substantial decline in *MFD* is already evident before around age 6, followed by stabilisation at lower values. Taken together, our data suggest that any residual regenerative capacity before the breakpoint could be inadequate to offset myofibre loss; once *MFD* stabilises at low levels, the exhausted reserve possibly channels disease evolution into patient‐specific remodelling characterised by the conventional area‐based markers. The earlier discussion of MFS and *MFD* dynamics around the 5‐ to 7‐year window further supports these interpretations of muscle regenerative capacity.

Clinical manifestations of DMD, including difficulties with stair climbing, a waddling gait and frequent falls, typically emerge between the ages of 3 and 5 years [1, 4, 21]. Additionally, contractures of the iliotibial bands, hip flexors and heel cords become apparent before the age of 6 years [24]. Notably, Zatz et al. reported that serum creatine kinase levels in patients with DMD are already elevated within the first year of life, progressively increasing until approximately age 6, after which they decline as a result of progressive muscle degeneration [38]. Furthermore, McDonald et al. reported a marked decline in performance on the 6‐min walk test around age 7 years in patients with DMD [39]. Similarly, Mayhew et al. demonstrated that steroid‐naïve boys with DMD aged 7 years exhibit significantly lower velocities in the rise‐from‐floor and 10‐m walk/run tests compared with those aged 4–6 years, reinforcing the functional downturn observed around ages 6–7 [40]. Thus, *MFD* dynamics may provide a histological framework for understanding these manifestations.

Our findings have important implications for therapeutic strategies. While multiple clinical guidelines recommend early corticosteroid initiation based on clinical conditions, such as early functional decline, the optimal timing remains uncertain because of the absence of pathological foundational evidence [1, 5, 12, 41]. A clustering‐based analysis by Fang et al. demonstrated that patients with DMD who received higher cumulative steroid exposure before age 6 exhibited slower disease progression compared with those who initiated corticosteroid therapy after this age [42]. In addition, recent randomised controlled trials investigating vamorolone, a steroid‐sparing anti‐inflammatory agent, demonstrated that treatment of steroid‐naïve patients with DMD aged 4–7 years yielded motor outcomes comparable to those of prednisone while mitigating detrimental effects on growth and bone health [43, 44], although this 4‐ to 7‐year enrolment window was not chosen based on the pathological natural history of DMD. These reports underscore the importance of identifying a pathology‐based therapeutic window using objective, reproducible and easily quantifiable biomarkers. Furthermore, several drug classes beyond corticosteroids have been evaluated or are under investigation for DMD therapy, each with distinct mechanisms of action and potentially different effects on *MFD*. For example, myostatin inhibitors, such as domagrozumab, primarily induce muscle fibre hypertrophy [45, 46]. These drugs may increase *MFA*, but their capacity to improve *MFD* through fibre number expansion is likely limited. Novel anti‐inflammatory agents, such as the NF‐*κ*B inhibitor edasalonexent, may slow *MFD* decline by suppressing early myofibre necrosis, as indicated by Phase 3 clinical trial results [47]. Exon‐skipping therapies and gene therapies aimed at stabilising the sarcolemma are expected to reduce necrosis–regeneration drivers, potentially slowing early *MFD* decline, consistent with evidence from dystrophin/microdystrophin restoration strategies [16, 23, 48]. Beyond the critical period, *MFD* stabilises at lower levels with a subtle downward trend, limiting the ability of our study design to predict therapeutic efficacy in later disease stages. Therefore, potential reversibility or delayed therapeutic effects on *MFD* cannot be inferred from this study alone. Additionally, qualitative changes in MFS and structure due to ongoing necrosis–regeneration cycles may follow *MFD* changes with a phase lag. These interpretations are preliminary based on established pharmacological mechanisms, existing clinical trial data and the detection characteristics of *MFD* demonstrated, highlighting the need for future prospective longitudinal studies.

We propose *MFD* as a candidate surrogate biomarker. In DMD drug development, several surrogates have supported accelerated approvals, yet each has faced debate regarding utility and validity. For example, eteplirsen (exon skipping) increased dystrophin in post‐treatment biopsies, measured by the percentage of immunohistochemically positive myofibres [11]. In the United States, the Food and Drug Administration (FDA) granted accelerated approval on this basis, but controversy followed regarding the prediction of clinical benefit and the comparability of methods [49, 50]. Microdystrophin has likewise been considered a surrogate candidate [48]; the FDA regards it as a ‘reasonably likely surrogate,’ but further correlation with clinical outcomes remains necessary [48]. Thus, two issues recur: whether a surrogate predicts clinical outcomes and whether the magnitude of change is clinically meaningful. As with existing surrogates, *MFD* will need standardised quantification, control of inter‐method and inter‐site variability, and reproducibility protocols. Additionally, for regulatory acceptance of *MFD*, three elements must be defined: Analytical Validity, Clinical Validity, and Context of Use [51, 52, 53]. Presumably, *MFD* would face similar barriers and scrutiny.

We believe that *MFD* also provides a histopathological ‘ground truth’ by defining muscle pathology as a density measure. Magnetic resonance imaging (MRI) as a surrogate endpoint is already being explored [54]. In the future, non‐invasive biomarkers, including computed tomography (CT) and MRI radiomics features [55, 56, 57] calibrated to the pathological reference standard of *MFD*, may prove highly promising. Therefore, the significance of this study lies not only in *MFD*'s potential as a surrogate endpoint, but also in its role as a benchmark for validating emerging surrogate biomarkers. Recent advances, particularly in tumour pathology, have demonstrated the value of novel image‐analysis methods, including CT radiomics, which have enabled applications such as characterising histopathological findings and identifying underlying genetic abnormalities from imaging features [55, 57]. Similar advanced CT/MRI methods could be applied to area‐independent histopathological parameters such as *MFD*, potentially enhancing their clinical and research applicability in neuromuscular diseases.

Muscle biopsy remains a critical tool in contemporary clinical trials, serving as both a primary and secondary outcome measure in the evaluation of new therapeutic strategies [11, 23, 48, 58]. Within this framework, *MFD* dynamics may provide an earlier, objective histological endpoint for assessing treatment efficacy and optimising intervention timing across diverse clinical settings. Given the simplicity of counting myofibres, *MFD* could serve as a straightforward, effective biomarker: whereas metrics such as the Ki‐67 labelling index and the mitotic count require qualitative judgements yet nonetheless achieve intraclass correlation coefficients greater than 0.90 in diagnostic pathology practice [59, 60], *MFD* is obtained by simply tallying every transverse myofibre with clearly identifiable contours. Although excluding all fibres intersecting the FOI boundary does not eliminate edge effects entirely, it provides a straightforward counting rule and supports accurate MFS measurements. Its simplicity and objectivity would facilitate seamless integration into routine pathology workflows and clinical trial endpoints, yielding clear practical advantages in real‐world settings.

To further clarify the methodological foundation underlying these advantages, we reiterate the definition of *MFD*. *MFD* includes all myofibres with identifiable contours: normal myofibres, internally nucleated fibres (*IntN*) and opaque fibres (*Opaque*), while excluding necrotic and regenerating fibres (*NFA* and *RFA*), which were quantified separately using area‐based metrics. This approach prevents systematic underestimation of *MFD* due to the exclusion of structurally preserved myofibres and distinguishes the cumulative dynamics of *MFD* from the transient dynamics of fibre degeneration and regeneration. *IntN* and *Opaque* reflect ongoing necrosis and regeneration cycles intrinsic to DMD pathology [30, 37]. However, as demonstrated in our data and additional plots (Figure S1), neither fibre type exhibited robust age‐dependent changes or identifiable breakpoints, even beyond the critical threshold of around 6 years of age. The consistent presence of these abnormal fibres suggests they represent a stable necrosis‐regeneration cycle and do not substantially influence the primary *MFD* dynamics observed in this study.

A further distinguishing feature of this study is that analysis of decades‐old archival biopsy specimens yielded these insights. Given the increasing emphasis on earlier corticosteroid and steroid‐sparing interventions described above, collecting such steroid‐naïve specimens would now be ethically challenging. In this study, the main analysis relied on the best‐preserved H&E, G‐T or M‐T section for each case. However, we also encountered severely faded ATPase‐stained specimens, such as the one shown in Figure 1a, which exemplified the actual extent of degradation. Nevertheless, after digital restoration we could still quantify *MFD* in these sections, and the discrepancies between the original and ATPase‐derived counts served as empirical estimates of measurement error (Δ*MFD* = 62 fibres/mm^2^) in our statistical analysis. Thus, the application of LUT‐based digital restoration enabled recovery of highly compromised histological material at minimal cost and rendered it amenable to further statistical validation. Additionally, Focus Stacking was employed to mitigate focal‐plane variations caused by uneven sectioning or coverslip mounting medium, thereby enabling accurate *MFD* quantification from well‐focused images even when histological preparations were suboptimal. These methodologies not only broaden the scope of routine pathology practice but also enable the revitalisation of archival specimens compromised by various adverse conditions, thereby expanding opportunities for histopathological research using archival datasets.

## 5 Study Limitations

This study has several limitations that warrant cautious interpretation.

First, our analysis was based on retrospective data with a small sample size. Although Monte Carlo power simulations indicated that our sample size was reasonably adequate for detecting the breakpoint, replication in larger, independent cohorts remains essential to confirm and refine these findings. Additionally, due to the uneven age distribution, the estimated breakpoint around 6 years should be considered an approximate range rather than a precise threshold. Furthermore, the cross‐sectional design precluded direct verification of longitudinal within‐subject trajectories of *MFD* decline.

Second, this investigation used archival biopsy samples collected between 1973 and 1984 from steroid‐naïve DMD patients at a single centre. Pre‐analytical variability—including preservation conditions, staining methods and mixed use of frozen and FFPE samples— was unavoidable. Although digital image restoration using LUT adjustment and Focus Stacking minimised fading and defocus artefacts, subtle biases related to these techniques could not be eliminated. Differences in muscle fibre shrinkage between frozen and FFPE preparations may also have affected myofibre‐related metrics, with myofibres in FFPE samples typically shrinking and thereby yielding lower *MFA* values and elevated *MFD* values. However, because only a small proportion of our samples were FFPE samples (Table S1), the overall influence of this limitation was likely modest. Furthermore, although potential batch effects arising from different staining protocols were only preliminarily evaluated on a pilot subset (see Data S2 and Table S3) and appeared negligible for area‐based measurements, subtle laboratory‐specific biases cannot be entirely excluded.

Third, the analyses focused primarily on histopathological features; thus, direct inferences regarding causality or underlying biological mechanisms could not be established.

Fourth, diagnosis in this historical cohort relied primarily on clinical and histopathological criteria, with limited genetic confirmation or dystrophin immunohistochemical validation, potentially introducing selection or diagnostic biases. Moreover, individual variability in genetic and clinical characteristics inherent in patients with DMD may influence the precise timing and nature of the identified transition phase, and the limited availability of detailed clinical data precluded comprehensive pathological–clinical correlation analyses. For example, correlating *MFD* with clinical severity (e.g., age at symptom onset, functional scores and ambulatory status) could enhance the translational value of our findings, highlighting the need for future studies with more comprehensive clinical data.

Fifth, all participants were steroid‐naïve. Contemporary clinical practices—including early corticosteroid treatment, steroid‐sparing agents and gene therapies—may significantly alter *MFD* dynamics compared to our archival cohort. Thus, the identified ‘pathological critical period around age 6 years’ reflects the natural history of disease pathology rather than a prescriptive treatment window. Prospective validation in contemporary treated cohorts remains necessary to confirm clinical relevance and generalisability. Additionally, standards of physiotherapy, orthotic use and complication management have evolved substantially, and their historical impact cannot be fully disentangled from our findings.

Finally, current clinical trials typically utilise biceps muscle biopsies, limiting the direct generalisability of our quadriceps‐derived results. The quadriceps and biceps muscles differ in the normal ranges of MFS [61], and findings from quadriceps biopsies cannot be extrapolated directly. Accordingly, the results of the logistic regression analysis, including age‐predictive *MFD* cut‐offs and theoretical therapeutic thresholds, should be interpreted with caution.

## 6 Conclusions

In conclusion, despite the inherent constraints associated with archival specimens, our methodological approach offers a novel perspective on early‐stage DMD and its clinical significance. The breakpoint around age 6 and the *MFD* dynamics established in our study may represent key benchmarks for assessing disease progression, with potential applications in clinical practice. However, validation through larger, systematically controlled studies is imperative. We propose that our findings support broader utilisation of archival specimens and help refine our understanding of the natural history of DMD, thereby informing future clinical management strategies for this severe, devastating neuromuscular disorder.

## Author Contributions

T.Y., Y.I., S.T. and Z.I.T. conceived and designed the study. T.Y., S.T. and Z.I.T. performed pathological diagnoses and semi‐quantitative analyses. T.Y. performed statistical analyses. S.T. and Z.I.T. supervised statistical analyses. T.Y. created the figures and tables. Y.I. collected relevant clinical literature and historical patient information. K.I., L.W., Y.O., M.T. and T.K. assisted with semi‐quantitative analyses. M.N. and M.I. organised archived patient records and facilitated data retrieval, performed data management and acquired internal funding. S.T. and Z.I.T. managed the overall project and provided comprehensive supervision of the study. All authors read and approved the final version of the article.

## Funding

This work was supported by a donation to the National Hospital Organization Hokkaido Medical Center for the project ‘Research on Quality of Life in Patients Undergoing Mechanical Ventilation’ (Y.I.).

## Ethics Statement

This study was approved by the Institutional Review Board of Hokkaido University Hospital (Approval number: 024‐0310). All procedures were conducted in accordance with the 1964 Declaration of Helsinki and its later amendments.

## Consent

Due to the retrospective observational nature of this study, the requirement for informed consent was waived by the Institutional Review Board.

## Conflicts of Interest

The authors declare no conflicts of interest.

## Supporting information


**Data S1:** Supporting Information.

**Data S2:** Supporting Information.

**Figure S1:** Scatter plots showing weak or non‐significant correlations between age at biopsy and four parameters reflecting abnormal muscle fibres (*Opaque*, *IntN*) and area‐based metrics of fibre degeneration/regeneration (*NFA*, *RFA*). The absence of significant age‐related trends or clear breakpoints in these variables suggests that these abnormal myofibres and transient degenerative/regenerative events do not significantly contribute to or confound the dynamics captured by *MFD*. Thus, the dynamics of *MFD* predominantly reflect a reduction in structurally preserved myofibres. (Abbreviations: NS, not significant; *Opaque*, opaque fibre percentage per myofibre density; *IntN*, internally nucleated fibre percentage per myofibre density; *NFA*, necrotic fibre area; *RFA*, regenerative fibre area; *MFD*, myofibre density.).

**Table S1:** The availability of sample.
**Table S2:** Variance analysis and post hoc test results across histological parameters.
**Table S3:** Stain‐based batch effect validation: comparison of MFA between paired H&E and G‐T Sections (n = 28, eData 2)*

## Data Availability

The datasets analysed during the current study and data not provided within this article because of space limitations are available from the corresponding author on reasonable request by qualified investigators for purposes of methodological replication and validation. Additionally, an accompanying Excel file containing the original dataset and the complete source code is provided as a supplementary file to facilitate reproducibility of the analyses. These materials are also publicly available at the following GitHub repository: https://github.com/tyamakadopatho2‐ops/Pathologist‐Repository‐for‐Open‐Science/tree/Neuropathol‐Appl‐Neurobiol‐2025. Supporting Information The supporting information includes Data S1 (original raw dataset), Data S2 (R script containing the complete source code used for all analyses and figure generation), Figure S1 with its accompanying legend (Figure S1 Legend; supplementary correlation plots referenced in Section 3) and eTable (a single document containing Tables S1–S3; supporting information tables cited in the main text). These files are provided to support transparency and reproducibility of the analyses reported in this manuscript.
