# Supplementary material for: Myofibre Density Reveals a Critical Threshold Around Age 6 in Steroid‐Naïve Duchenne Muscular Dystrophy: A Retrospective Observational Study
Source: Neuropathol Appl Neurobiol. 2026 Mar 18;52(2):e70068. doi: 10.1111/nan.70068 (PMC12997517; doi:10.1111/nan.70068)
Supplement: Supplementary file 4 — Table S1: The availability of sample. Table S2: Variance analysis and post hoc test results across histological parameters. Table S3: Stain‐based batch effect validation: comparison of MFA between paired H&E and G‐T Sections (n = 28, eData 2)* [file NAN-52-e70068-s004.docx]

| **participant** | **Frozen H&E** | **Frozen G-T** | **FFPE H&E** | **FFPE M-T** | **ATPase** |
| --- | --- | --- | --- | --- | --- |
| 1 | **○** | **○** | **×** | **×** | **○** |
| 2 | **○** | **○** | **×** | **×** | **×** |
| 3 | **×** | **×** | **○** | **○** | **×** |
| 4 | **○** | **○** | **○** | **×** | **○** |
| 5 | **○** | **○** | **×** | **×** | **×** |
| 6 | **×** | **×** | **○** | **○** | **×** |
| 7 | **○** | **○** | **×** | **×** | **○** |
| 8 | **○** | **○** | **×** | **×** | **×** |
| 9 | **○** | **○** | **○** | **○** | **×** |
| 10 | **○** | **○** | **×** | **×** | **×** |
| 11 | **○** | **○** | **○** | **○** | **×** |
| 12 | **○** | **○** | **×** | **×** | **○** |
| 13 | **○** | **○** | **×** | **×** | **○** |
| 14 | **×** | **×** | **×** | **○** | **×** |
| 15 | **○** | **○** | **×** | **×** | **×** |
| 16 | **○** | **○** | **×** | **○** | **×** |
| 17 | **×** | **×** | **○** | **×** | **×** |
| 18 | **○** | **○** | **×** | **×** | **×** |
| 19 | **○** | **○** | **×** | **×** | **○** |
| 20 | **○** | **○** | **×** | **×** | **○** |
| 21 | **○** | **○** | **×** | **×** | **○** |
| 22 | **○** | **○** | **×** | **×** | **×** |
| 23 | **○** | **○** | **×** | **×** | **×** |
| 24 | **○** | **×** | **×** | **×** | **×** |
| 25 | **○** | **○** | **×** | **×** | **×** |
| 26 | **○** | **○** | **×** | **×** | **×** |
| 27 | **○** | **○** | **×** | **×** | **×** |
| 28 | **○** | **○** | **○** | **○** | **×** |
| 29 | **○** | **○** | **×** | **×** | **○** |
| 30 | **×** | **○** | **×** | **×** | **○** |
| 31 | **○** | **○** | **×** | **×** | **×** |
| 32 | **○** | **○** | **×** | **×** | **○** |
| 33 | **○** | **○** | **×** | **×** | **○** |
| 34 | **×** | **○** | **×** | **×** | **○** |
| 35 | **○** | **○** | **×** | **×** | **×** |
| 36 | **○** | **○** | **×** | **×** | **×** |
| 37 | **×** | **○** | **×** | **×** | **×** |
| 38 | **×** | **○** | **×** | **×** | **○** |

**eTable 1: The availability of sample**

(Abbreviations: FFPE, formalin-fixed paraffin-embedded; H&E, haematoxylin-eosin; G-T, Gomori Trichrome; M-T, Masson Trichrome; ATPase, actomyosin adenosine triphosphatase.)

This table details the availability of specimen preparations (frozen and FFPE) and staining techniques (H&E, G-T, M-T, and ATPase) for each patient.

○: available (samples used in the primary analysis are highlighted in blue).

×: severe fading, insufficient tissue quantity, or lost.

**eTable 2: Variance analysis and post-hoc test results across histological parameters**

|  | **Comparison** | **Effect size [95% CI]** | **Mean difference [95% CI]** | ***p* value**^‡^ | **Nreq^§^** |
| --- | --- | --- | --- | --- | --- |
| ***Mean**** | 1–5 vs 5–7.5 years | 0.61 [0.22 to 0.92] | 403.72 [145.14 to 662.29] | <0.01 | 11.74 |
|  | 1–5 vs 7.5–11 years | 0.80 [0.49 to 1.00] | 620.75 [93.48 to 1148.02] | <0.01 | 5.88 |
|  | 5–7.5 vs 7.5–11 years | 0.09 [−0.38 to 0.57] | 217.03 [−345.70 to 779.77] | 0.55 | 602.95 |
| ***Sd**** | 1–5 vs 5–7.5 years | 0.73 [0.38 to 0.97] | 414.10 [180.25 to 647.95] | <0.01 | 7.43 |
|  | 1–5 vs 7.5–11 years | 0.88 [0.65 to 1.00] | 861.90 [288.90 to 1434.90] | <0.0001 | 4.38 |
|  | 5–7.5 vs 7.5–11 years | 0.30 [−0.16 to 0.73] | 447.80 [−154.31 to 1049.91] | 0.18 | 53.58 |
| ***Cov**** | 1–5 vs 5–7.5 years | 0.48 [0.007 to 0.89] | 0.16 [−0.019 to 0.33] | 0.039 | 19.74 |
|  | 1–5 vs 7.5–11 years | 0.83 [0.49 to 1.00] | 0.35 [0.12 to 0.59] | <0.001 | 5.24 |
|  | 5–7.5 vs 7.5–11 years | 0.40 [−0.06 to 0.79] | 0.20 [−0.03 to 0.42] | 0.064 | 29.75 |
| ***MFD*^†^** | 1–5 vs 5–7.5 years | −1.89 [−3.06 to −1.01] | −431.25 [−641.04 to −221.46] | <0.0001 | 6.35 |
|  | 1–5 vs 7.5–11 years | −2.71 [−3.97 to −1.89] | −573.52 [−791.87 to −355.16] | <0.0001 | 3.75 |
|  | 5–7.5 vs 7.5–11 years | −0.88 [−1.69 to −0.26] | −142.27 [−352.06 to 67.52] | 0.23 | 21.43 |
| ***MFA*^†^** | 1–5 vs 5–7.5 years | −1.05 [−2.08 to −0.33] | −11.78 [−24.75 to 1.20] | 0.12 | 15.39 |
|  | 1–5 vs 7.5–11 years | −1.36 [−2.54 to −0.65] | −18.74 [−32.25 to −5.24] | 0.015 | 9.52 |
|  | 5–7.5 vs 7.5–11 years | −0.47 [−1.42 to 0.33] | −6.97 [−19.94 to 6.01] | 0.40 | 72.31 |
| ***Fat**** | 1–5 vs 5–7.5 years | 0.36 [−0.10 to 0.78] | 1.50 [−0.69 to 3.70] | 0.15 | 37.6 |
|  | 1–5 vs 7.5–11 years | 0.75 [0.36 to 1.00] | 5.28 [2.04 to 8.52] | <0.01 | 6.98 |
|  | 5–7.5 vs 7.5–11 years | 0.57 [0.12 to 0.94] | 3.78 [0.12 to 7.44] | 0.026 | 13.83 |
| ***CFA*^†^** | 1–5 vs 5–7.5 years | 0.99 [0.31 to 1.92] | 10.19 [−1.79 to 22.17] | 0.16 | 16.89 |
|  | 1–5 vs 7.5–11 years | 1.04 [0.31 to 2.16] | 13.19 [0.72 to 25.66] **^¶^** | 0.11**^¶^** | 15.52 |
|  | 5–7.5 vs 7.5–11 years | 0.21 [−0.62 to 1.13] | 3.00 [−8.98 to 14.98] | 0.81 | 336.47 |

(Abbreviations: CI, confidence interval; *CFA*, connective/fibrotic tissue area*; Cov*, coefficient of variation of myofibre size; *Fat*, fatty degeneration area; *Mean*, mean myofibre size; *MFA*, myofibre area; *MFD*, myofibre density; *Sd*, standard deviation of myofibre size; FDR, false-discovery-rate.)

***** Cliff's *δ* for variables violating normality assumptions (*Mean*, *Sd*, *Cov*, and *Fat*).

**^†^** Hedges' *d* for normally distributed variables with equal variances (*MFD*, *MFA*, and *CFA*).

^‡^ FDR-adjusted *p* values

**^§^** Nreq—defined as the per-group sample size required to detect the observed effect with 80% power (*α* = 0.05, two-sided)—was calculated with Hedges' *d* for ^†^ variables and Cliff's *δ* for ***** variables.

**^¶^** CI excludes zero, but FDR-adjusted *p* = 0.11 (>0.05); hence deemed not significant.

**eTable 3: Stain-based batch effect validation: comparison of *MFA* between paired H&E and G-T Sections (n = 28, eData 2)***

| **Panel** | **Statistic type** | **Point estimate** | ***p* / BF** | **Interpretation** |
| --- | --- | --- | --- | --- |
| A. Frequentist tests | Wilcoxon signed rank **^a^** | — | *p* = 0.93 | — |
|  | Paired *t* **^a^**  (*Df* = 27) | — | *p* = 0.91 | means equivalent |
|  | Hedges’ *d* | 0.03  (CI –0.48, 0.57)**^b^** | — | negligible effect |
| B. Bayesian tests | JZS BF**^c^** | — | BF_10_ = 0.101 | moderate evidence for *H*_0_ |
|  | Posterior *δ* | 0.12  (CI –0.26, 0.49) | — | concordant |
|  | ROPE mass**^d^** | 82%  within ±0.30 | — | practical equivalence |
| C. Reliability / batch | Pearson *r* **^e^** | 0.97  (CI 0.97, 0.99) | *p* <0.001 | high concordance |
|  | Specimen τ^2^ **^f^** | 165.36 → 165.30  Δτ^2^ = 0.03% | — | stain effect  negligible |

(**Abbreviations**: BF, Bayes factor; CI, confidence interval; G-T, Gomori trichrome; H&E, haematoxylin-eosin; JZS, Jeffreys–Zellner–Siow; *MFA*, myofibre area; ROPE, Region of Practical Equivalence.)

***** n = 28 paired sections from identical specimens; no missing data (see also **eData 2**).

**^a^** Two-sided tests; significance threshold *p* <0.05.

**^b^** 95% CI for Hedges’ *d* obtained via 5,000-resample bias-corrected and accelerated bootstrap.

**^c^** JZS BF calculated with default Cauchy prior scale *r* = √2 ⁄ 2. BF_10_ <1 favours the null (*H*_0_); interpretive labels.

**^d^** ROPE; interval |*δ*| <0.30 corresponds to a “small” effect size threshold.

**^e^** Pearson product-moment correlation quantifies absolute agreement of *MFA* between stains; CI via Fisher *z* transformation.

**^f^** τ^2^ denotes between-specimen random-effects variance from linear mixed-effects models. Δτ^2^ expresses the percentage reduction after including “stain” as a fixed effect; a reduction <5 % is interpreted as negligible batch influence.
